# Short-term treatment of CIDP with efgartigimod: a case series in China

**DOI:** 10.3389/fimmu.2025.1533167

**Published:** 2025-05-01

**Authors:** Chong Sun, Jianian Hu, Yanyin Zhao, Yongsheng Zheng, Quanhua Meng, Sushan Luo, Kai Qiao, Jian Sun, Jiahong Lu, Jie Lin, Chongbo Zhao

**Affiliations:** ^1^ Department of Neurology, Huashan Hospital, Fudan University, Shanghai, China; ^2^ National Center for Neurological Disorders, Fudan University, Shanghai, China; ^3^ Huashan Rare Disease Center, Shanghai Medical College, Fudan University, Shanghai, China; ^4^ People’s Hospital of Deyang City, Deyang, Sichuan, China

**Keywords:** chronic inflammatory demyelinating polyradiculoneuropathy (CIDP), efgartigimod, short-term, treatment, real-world study, China

## Abstract

**Objective:**

Chronic inflammatory demyelinating polyradiculoneuropathy (CIDP) is a type of autoimmune neuropathy with treatment challenges due to the limitations of standard of care therapies. Efgartigimod, a neonatal Fc receptor antagonist, has shown potential in treating antibody-mediated disorders including CIDP (ADHERE study), but real-world studies on the application of efgartigimod in CIDP are still lacking. This study aimed to evaluate the short-term efficacy and safety of efgartigimod in five patients with CIDP in China.

**Methods:**

Clinical effectiveness was assessed using the Inflammatory Neuropathy Cause and Treatment (INCAT) disability scale, Inflammatory Rasch-built Overall Disability Scale (IRODS), Medical Research Council (MRC) sum score (0–60), grip strength, Neuropathy Impairment Score (NIS), and 3-m Time Up and Go Test (TUG). Safety was evaluated by monitoring adverse events and measuring white blood cell count, serum albumin concentration, and plasma IgG concentration. Peripheral CD4^+^ T and CD19^+^ B lymphocytes were measured before and after efgartigimod treatment.

**Results:**

All five (100%) patients responded to efgartigimod treatment, with four (80%) meeting predefined effectiveness criteria within 8 weeks. The average reduction rate in total IgG was 43%. Adverse events were minimal, with one patient experiencing transient diarrhea, and no aggravation of pre-existing conditions was noted.

**Interpretation:**

Efgartigimod demonstrates promising efficacy and safety for short-term treatment of CIDP, offering a potential alternative therapy. This study provides valuable evidence from the real-world application of efgartigimod in CIDP, and the results indicate further research is warranted.

## Introduction

Chronic inflammatory demyelinating polyradiculoneuropathy (CIDP) is a type of autoimmune neuropathy presenting with progressive symmetric proximal and distal muscle weakness, sensory loss, and decreased or absent deep tendon reflexes with progression over 8 weeks. The reported incidence and prevalence rates for CIDP have varied from 0.2–1.6 and 0.8–8.9 per 100,000 individuals, with a higher occurrence observed in males and older adults (1). The standard of care therapies for CIDP, including intravenous immunoglobulin (IVIg) administration, corticosteroid therapy, and plasma exchange, have been proven effective. However, there are still several limitations, such as chronic side effects, reliance on plasma donations, and considerable economic burden (2).

Although our understanding of CIDP pathogenesis remains incomplete, significant progress has been made in recent years. Cell-mediated pathology involving CD4^+^ T cells, CD8^+^ T cells and macrophages was demonstrated in CIDP. These immune cells infiltrate the endoneurium, disrupt the blood-nerve barrier, and activate the release of cytokines and chemokines, leading to nerve damage (3). Complement is also involved in the pathogenesis of CIDP, as it has been identified in the serum, cerebrospinal fluid samples, and sural nerve biopsy specimens from patients with CIDP. Complement appears to promote macrophage-mediated demyelination (4). Autoantibodies against peripheral nerve molecules, such as gangliosides or proteins of the nodes of Ranvier, have an important role in the pathogenesis of CIDP, causing demyelination and axonal damage (5). This is supported by the response to plasma exchange and B-cell depletion therapy.

The neonatal Fc receptor (FcRn), a receptor for the crystallizable fragment (Fc) of IgG, prolongs IgG half-life by recycling and preventing lysosomal degradation in a pH-dependent manner (6). Blocking FcRn and inhibiting its function facilitates the degradation of IgG antibodies, offering a promising, attractive, and novel targeted treatment strategy for immune-mediated disorders (7). Efgartigimod is a human IgG1-derived Fc fragment that binds to human FcRn, preventing IgG recycling and increasing its degradation without impacting the albumin levels or those of other immunoglobulins (8). Efgartigimod is the first FcRn antagonist to receive approval for clinical use and is now available in multiple countries for the treatment of myasthenia gravis (MG) and CIDP (9). Efgartigimod has also demonstrated favorable treatment efficacy and safety in real-world studies of patients with MG, Guillain-Barré syndrome, and Stiff-Person syndrome (10–12). Recently, the pivotal ADHERE study on the use of efgartigimod for treating CIDP showed promising outcomes (13). However, real-world experience on the application of efgartigimod in CIDP is still lacking.

In the present study, we evaluated the short-term efficacy and safety of efgartigimod in a case series of five patients with CIDP, aiming to provide the first data from a real-world experience in China.

## Methods

### Patients

This prospective observational case series was conducted in a single center from October to December 2023. All five CIDP patients included in this study met the 2021 European Academy of Neurology and Peripheral Nerve Society (EAN/PNS) criteria for CIDP diagnosis (14). All patients had persistent immunologically active CIDP as evidenced by chronic progression with superimposed relapses and change in disease activity related to treatment (15). Patients with antibodies against nodal/paranodal cell adhesion molecules (contactin-1 [CNTN1], neurofascin-155 [NF155], contactin-associated protein 1 [Caspr1], or neurofascin isoforms NF140/186) were excluded. The cell-based assay was performed for the initial screening of anti-nodal/paranodal antibodies, as detailed in our previous study (16). Immunofixation electrophoresis of all patients was negative. This study was conducted in accordance with the Declaration of Helsinki ethical guidelines and was approved by the ethics committee of Huashan Hospital, Fudan University (2022–849–1). All patients gave their written informed consent for participation in the study.

### Treatment

In this cohort, efgartigimod was administered intravenously at a dosage of 10 mg/kg. The regimen was tailored to each individual patient rather than adhering to a four-dose cycle (9), with decisions regarding subsequent doses based on each patient’s weekly response to the medication.

### Evaluation of clinical effectiveness

Clinical evaluations were conducted at baseline and weekly for a total of 8 weeks. Results for the Inflammatory Neuropathy Cause and Treatment (INCAT) disability scale, the Inflammatory Rasch-built Overall Disability Scale (IRODS), the Medical Research Council (MRC) sum score (0–60), grip strength (tested using a Martin Vigorimeter), the Neuropathy Impairment Score (NIS), and the 3-m Time Up and Go Test (TUG) were collected to evaluate clinical improvement prospectively. Response to efgartigimod was defined as improvement in any of the above clinical scales. The effectiveness of efgartigimod was defined by the fulfillment of any of the following conditions: 1-point decrease in the INCAT, 4-point increase in the IRODS, 4-point increase in the MRC sum score, or 8-kPa increase in grip strength compared to baseline (17). The TUG and NIS were used as supplementary indicators, with conditions of a 0.5s decrease in the TUG or an 8-point decrease in the NIS (18) to support a clinical improvement.

### Evaluation of safety

All adverse events during and after efgartigimod treatment were reported, including symptoms of infection. White blood cell count and serum albumin were measured at the initial administration of efgartigimod and at the last follow-up. The concentration of total IgG in plasma was measured by an immunoturbidimetric assay. The reduction rate of IgG was calculated relative to the baseline concentration.

### Flow cytometry

Flow cytometric immunophenotyping was performed to identify peripheral CD4^+^ T-cell and CD19^+^ B-cell subsets in patients’ blood samples before and after efgartigimod treatment. The frequency of CD4^+^ T-cell subsets was assessed as follows: CD25^hi^CD127^dim^ regulatory T cells (Tregs); CXCR3^+^ CCR6^–^ T helper 1 (Th1) cells; CXCR3^–^ CCR6^–^ Th2 cells; and CXCR3^–^ CCR6^+^ Th17 cells. The frequency of CD19^+^ B-cell subsets was assessed as follows: CD38^+^ CD138^+^ plasma cells, CD24^-^CD27^hi^CD38^hi^CD20^-^ plasmablasts, CD27^+^ memory B cells, IgD^+^ CD27^–^ naïve B cells, IgD^+^ CD27^+^ unswitched memory B cells, IgD^–^ CD27^+^ switched memory B cells, and CD24^hi^CD38^hi^ regulatory B cells (Bregs). We followed the protocol described in our previous study (19, 20).

### Statistical analysis

Categorical data were presented as frequencies and percentages. Continuous data were expressed as means and standard deviations (SD).Although the sample size was small, previous studies have shown that age, disease duration and clinical scores tend to be normally distributed in CIDP (13, 21). Statistical analyses were performed using GraphPad Prism 9 (GraphPad Software, LLC).

## Results

### Demographic and clinical characteristics of CIDP patients

The demographic and clinical characteristics of the five patients are presented in **Table 1**. All five patients with CIDP were included in the final analysis, including two males and three females. The mean age of the cohort was 41.2 years (range, 18–72 years). The mean duration of illness was 21.4 months (range, 5–54 months). The onset was chronic in four patients and acute in one. All patients had limb weakness, impaired superficial and vibration sensation, and abnormal tendon reflexes. On electrophysiologic examination, four patients exhibited sensorimotor demyelinating polyneuropathy with axonal damage, while one patient showed motor demyelinating polyneuropathy. Cerebrospinal fluid (CSF) protein levels were markedly elevated across all five cases, with a mean total protein concentration of 1156 mg/L (range, 567–1830 mg/L). Comorbidities included Sjögren’s syndrome in one patient and a history of recurrent urinary tract infections in another patient. All patients had received at least two of the standard of care therapies for CIDP. The effectiveness of prior therapy was defined according to previously reported criteria (17). Notably, three of the five cases were classified as refractory, with limited or no response to two or more standard of care therapies.

**Table 1:** Demographic and clinical characteristics of CIDP patients.

	P1	P2	P3	P4	P5
Age (years)	18	67	21	28	72
Gender	Male	Female	Female	Male	Female
Duration (months)	12	14	22	54	5
Onset type	Chronic	Chronic	Chronic	Chronic	Acute
CIDP type	Typical	Motor	Typical	Typical	Typical
Limb weakness	UL+LL	LL	UL+LL	UL+LL	UL+LL
Sensory abnormality					
Superficial sensation	LL	Normal	UL+LL	UL+LL	UL+LL
Vibration sensation	LL	LL	Normal	UL+LL	UL+LL
Abnormal tendon reflexes	UL+LL	LL	UL+LL	UL+LL	UL+LL
Electrophysiologic examination	Sensorimotor demyelinating polyneuropathy with axonal damage	Motor demyelinating polyneuropathy	Sensorimotor demyelinating polyneuropathy with axonal damage	Sensorimotor demyelinating polyneuropathy with axonal damage	Sensorimotor demyelinating polyneuropathy with axonal damage
CSF protein level (mg/L)	993	991	1830	567	1198
Comorbidities	None	Sjögren’s syndrome	None	None	Urinary tract infection
Prior treatment					
IVIg	Partially effective	Effective	Ineffective	Partially effective	Partially effective
Plasma exchange	None	None	Effective	Partially effective	Partially effective
Corticosteroids	Effective	Effective	Partially effective	Partially effective	Partially effective
Rituximab	None	None	Ineffective	Ineffective	None
Dose of efgartigimod	10 mg/kg	10 mg/kg	10 mg/kg	10 mg/kg	400 mg for the first two injections, then increased to 800 mg
Dosing regimen of efgartigimod	Once a week for 2 weeks	Once a week for 2 weeks	Once a week for 4 weeks, thereafter, once a month	Once a week for 8 weeks	Once a week for 4 weeks
Concomitant medications at start of efgartigimod treatment	Mycophenolate mofetil, corticosteroids	Cyclophosphamide, total glucosides of paeony, mycophenolate mofetil, corticosteroids	Corticosteroids	Corticosteroids	None
Concomitant medications at last follow-up	Mycophenolate mofetil, corticosteroids, rituximab	Cyclophosphamide, total glucosides of paeony, mycophenolate mofetil, corticosteroids	Corticosteroids	Corticosteroids	Rituximab

LL, lower limb; UL, upper limb; CSF, cerebrospinal fluid; IVIg, intravenous immunoglobulin.

### Treatment completion

The regimen of efgartigimod treatment was tailored to each individual patient. Patients 1 and 2 exhibited mild symptoms and received weekly treatments for 2 weeks. Patient 3 presented with more severe symptoms and received weekly treatments for 4 weeks, followed by a maintenance dose every 4 weeks. Patient 4, who presented with a longer disease course, more severe symptoms, and poor response to standard of care and rituximab, received a weekly dosing schedule for 8 weeks. Patient 5, who had a history of recurrent urinary tract infections and abnormal results on routine urine testing at baseline, initially received a reduced dose (400 mg). Subsequent dose adjustments to 800 mg were made after observation of normal blood and urine test results as well as severe symptoms. Due to the severe symptoms and slower response experienced in this patient, combination treatment of efgartigimod with rituximab was applied, starting with weekly doses of efgartigimod for the first 4 weeks and introduction of rituximab in the sixth week.

### Clinical effectiveness of efgartigimod in CIDP

**Figure 1:**
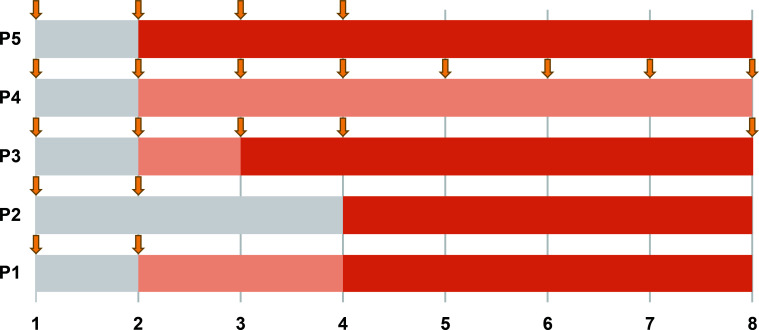
Timeline of improvement with efgartigimod treatment for all five patients. Each row represents a patient, and the numbers along the bottom represent time in weeks. The phase post-medication where no clinical response was observed is shaded in gray; the phase where a clinical response occurred but treatment was not yet effective is marked by light orange; and the phase of an effective response is shown in orange. Downward arrows mark the timepoints of efgartigimod administration.

In this cohort, all five (100%) patients showed a response to efgartigimod treatment, and the treatment was considered effective in four patients (80%) according to the predefined criteria (**Table 2**). Overall, improvements were observed in all clinical scales in all patients, of whom four achieved clinical responses at week 1 and four exhibited treatment effectiveness at week 4 (**Figure 1**).

**Table 2:** Evaluation of clinical effectiveness and safety of efgartigimod treatment.

	Patient 1		Patient 2		Patient 3		Patient 4		Patient 5	
	Start	Last	Start	Last	Start	Last	Start	Last	Start	Last
INCAT	2	1	2	1	3	0	8	8	9	9
IRODS	43	45	43	46	28	48	1	1	0	0
NIS	34	30	20	15	80	20	149.5	144	180	175
MRC	60	60	58	60	46	60	27	30	0	7
Grip strength (right/left, kPa)	71.3/73.3	72/72	40.7/40.3	50.74/40	15.3/20	76.7/70.3	0/0	0/0	0/0	0/0
TUG (s)	9.5	8.5	8.5	8.5	Unable to complete	8	Unable to complete	Unable to complete	Unable to complete	Unable to complete
IgG (g/L)	9.96	6.69	8.75	6.36	7.24	4.38	8.45	2.96	8.63^*^	4.3
WBC count (*10^9/L)	6.72	7.16	4.11	6.36	8.57	7.65	8.45	7.28	6.55	5.6
Serum albumin (g/L)	43	47	43	48	43	47	40	48	37	47

*This IgG level was measured prior to the second administration of efgartigimod because the baseline value was not available. “Start” indicates the timepoint of the initial administration of efgartigimod, and “Last” indicates the timepoint of last follow-up. INCAT, Inflammatory Neuropathy Cause and Treatment disability scale; IRODS, Inflammatory Rasch-built Overall Disability Scale; MRC, Medical Research Council sum score; NIS, Neuropathy Impairment Score; TUG, Time Up and Go Test; WBC, white blood cell. Response to efgartigimod was defined as improvement in any of the clinical scales. The effectiveness of efgartigimod was defined by the fulfillment of any of the following conditions: 1-point decrease in the INCAT, 4-point increase in the IRODS, 4-point increase in the MRC, or 8-kPa increase in grip strength compared to baseline. The TUG and NIS were used as supplementary indicators, with conditions of a 0.5s decrease in the TUG or an 8-point decrease in the NIS to support a clinical improvement.

Patients 1 and 2, who had comparatively milder symptoms, demonstrated smaller changes in scale scores but met the effectiveness criteria based on improvement on the INCAT scale. Patient 3 exhibited a dramatic response to treatment, showing marked improvements across all scales and meeting the effectiveness criteria. Patients 4 and 5, who presented with more severe symptoms, did not show changes in INCAT and IRODS scores. However, they exhibited sustained improvements in NIS and MRC scores, with Patient 5 achieving clinical effectiveness in the MRC sum score. Limited effectiveness of efgartigimod treatment was observed in Patient 4, who had the longest disease course of 54 months.

**Figure 2:**
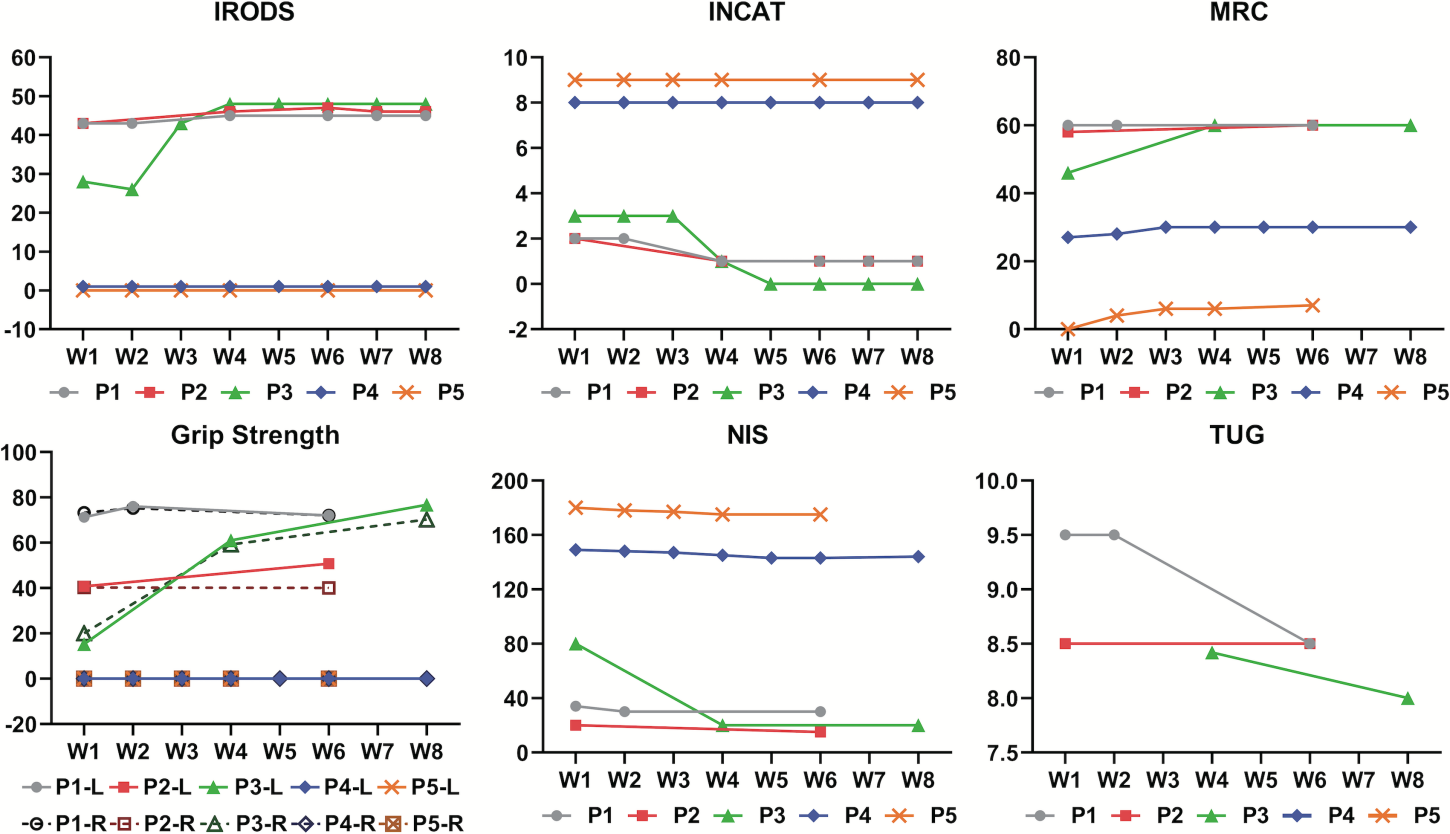
Spectrum of responses to efgartigimod evaluated by various clinical scales among all five patients over 8 weeks. W1 to W8 on the horizontal axis represents the first week to the eighth week of efgartigimod treatment. The figure for grip strength includes scores for the left and right hands of each patient, which are indicated by letters L and R, respectively, in the legend entry. INCAT, Inflammatory Neuropathy Cause and Treatment disability scale; IRODS, Inflammatory Rasch-built Overall Disability Scale; MRC, Medical Research Council sum score; NIS, Neuropathy Impairment Score; TUG, Time Up and Go Test. INCAT, IRODS, MRC and NIS were measured in scores. Grip strength was measured in kilopascals (kPa).TUG score was measured in seconds.

In this case series, the response to efgartigimod was evaluated using various clinical scales (**Supplementary Table**), which revealed a range of responses among the patients (**Figure 2**). In the INCAT, three patients showed responses and met the criterion for effectiveness, while the two severely affected patients showed no response. In the IRODS, three patients showed a response of whom only one met the effectiveness criterion, while the other two did not due to their relative high baseline scores. The MRC results were promising, with four patients responding and two showing effective outcomes. However, Patient 1 had a maximum score at baseline, and thus, could not show a response on this scale. Regarding grip strength, three patients showed responses, with two achieving improvement. Notably, in the NIS score, all five patients responded, but the response indicated treatment effectiveness in only one patient.

### Safety profile of efgartigimod in CIDP

**Figure 3:**
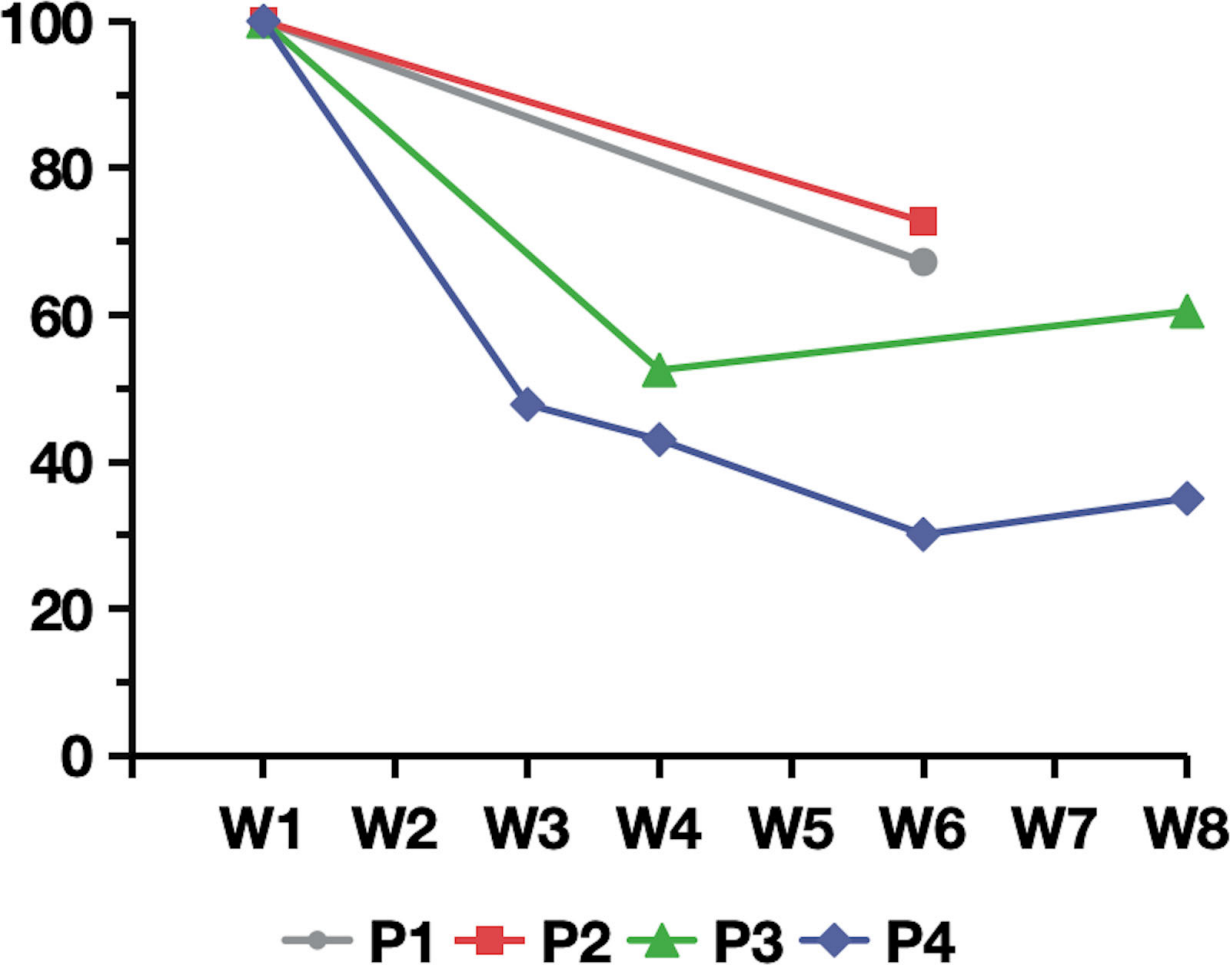
Percentage of plasma IgG relative to baseline following efgartigimod treatment for patient 1 to patient 4. W1 to W8 on the horizontal axis represents the first week to the eighth week of efgartigimod treatment.

In the safety evaluation, total IgG concentrations in plasma were measured by an immunoturbidimetric assay. All patients demonstrated a reduction in the total IgG level.The average decrease in IgG compared to baseline was 40.45% in 4 patients (range, 27.5%–65%) (**Figure 3**). Regarding adverse events, only one patient experienced a transient episode of diarrhea, which resolved spontaneously. The other four patients reported no adverse symptoms or discomfort. Patient 5, who had a history of recurrent urinary tract infections and abnormal baseline results on routine urine testing, experienced no aggravation of the infection during efgartigimod treatment, and routine urine test results returned to normal. White blood cell counts remained within the normal range for all five patients. Additionally, the serum albumin levels in all five patients did not decrease but showed a slight increase (**Table 2**).

### Concomitant medications

At the initiation of efgartigimod treatment, four of the five patients had been undergoing concomitant therapies for more than 6 months, including steroids, mycophenolate mofetil, cyclophosphamide, and total glucosides of paeony. Rituximab was prescribed for Patients 1 and 5 as maintenance treatments at week 6, at which point both patients had achieved clinical effectiveness. Patient 2 maintained the same concomitant therapy, and Patient 3 and 4 were taking a reduced dosage of oral steroids (**Table 1**).

### Effect of efgartigimod on T and B cells

**Figure 4:**
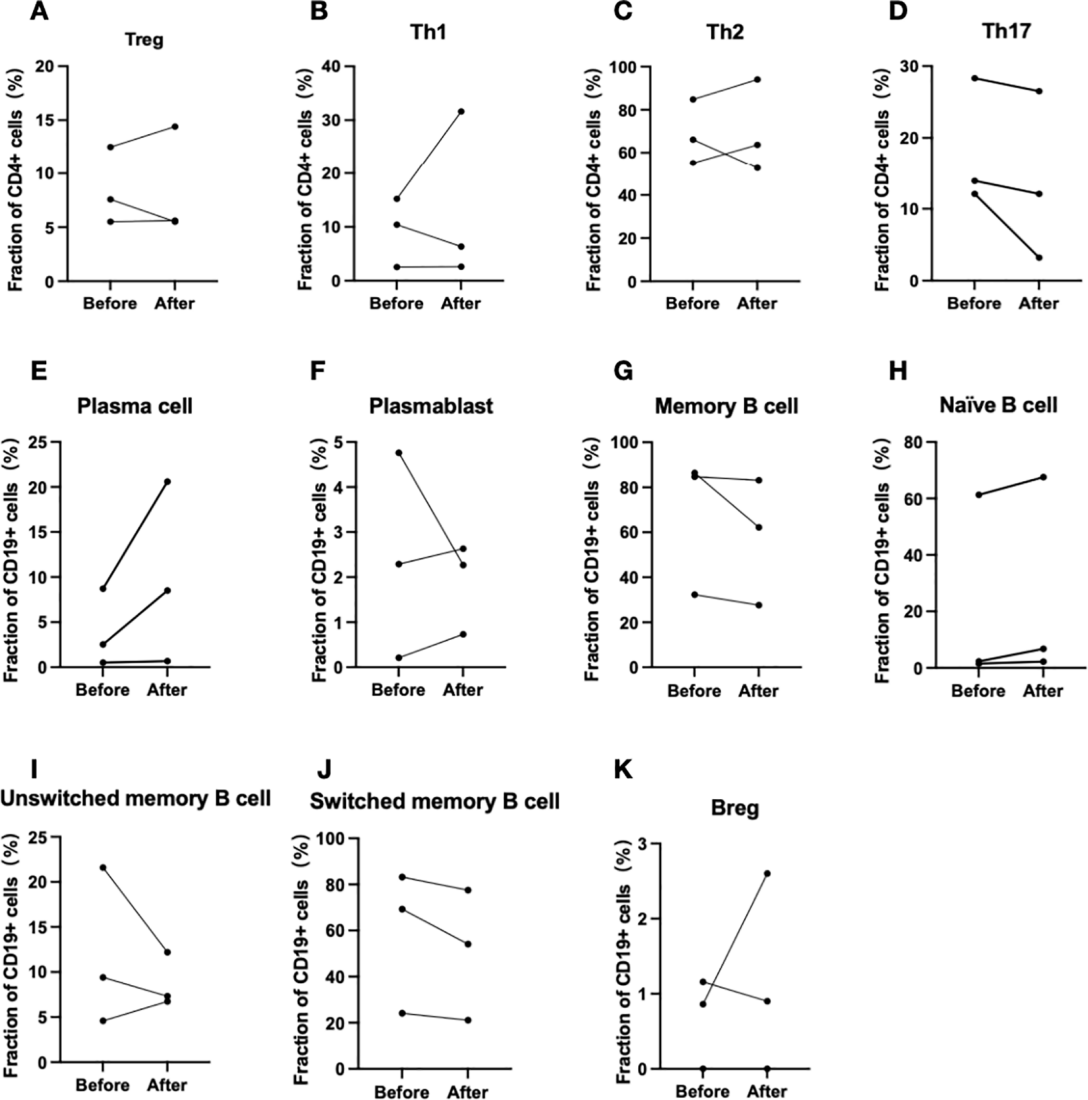
Changes in T and B lymphocyte subpopulations before and after efgartigimod administration(Patient 3, 4 and 5). **(A–D)** Changes in T lymphocyte subpopulations, and **(E–K)** corresponding alterations in B lymphocyte subpopulations.

Flow cytometric analysis was conducted on peripheral blood T and B lymphocytes from three patients (Patients 3, 4, and 5) before and after treatment (**Table 3**). The results indicated a consistent trend across Th17 cells, memory B cells, switched memory B cells, naïve B cells, and plasma cells. Specifically, the proportion of Th17 cells, memory B cells, and switched memory B cells decreased following treatment, while the proportions of naïve B cells and plasma cells increased (**Figure 4**).

**Table 3:** Changes in peripheral CD4^+^T and CD19^+^B profile of CIDP patients before and after efgartigimod treatment.

	Before treatment (n=3)	After treatment (n=3)
Treg, %in CD4+T	8.53 ± 3.60 (5.50-12.50)	8.51 ± 5.10 (5.51-14.40)
Th1, %in CD4 +T	9.38 ± 6.40 (2.54-15.20)	13.52 ± 15.77 (2.61-31.60)
Th2, %in CD4+T	68.67 ± 15.02 (55.10-84.80)	70.13 ± 21.39 (52.70-94.00)
Th17, %in CD4 +T	18.10 ± 8.88 (12.10-28.30)	13.94 ± 11.75 (3.21-26.50)
Plasma, %in CD19 +B	3.92 ± 2.78 (0.51-8.72)	9.93 ± 10.04 (0.68-20.60)
Plasmablast, %in CD19 +B	2.42 ± 2.78 (0.21-4.76)	1.87 ± 1.01 (0.73-2.63)
Memory B, %in CD19 +B	67.80 ± 30.76 (32.30-86.40)	57.63 ± 28.03 (27.60-83.10)
Naïve B, %inCD19 +B	21.70 ± 34.30 (1.53-61.30)	25.54 ± 36.50 (2.25-67.60)
Unswitched memory B, %in CD19 +B	11.87 ± 8.77 (4.59-21.60)	8.76 ± 3.00 (6.74-12.20)
Switched memory B, %in CD19 +B	58.87 ± 30.90 (24.10-83.20)	50.90 ± 28.34 (21.10-77.50)
Breg, %in CD19 +B	0.67 ± 0.60 (0.00-1.60)	1.17 ± 1.32 (0.00-2.60)

Data are mean ± SD (range). Treg, regulatory T; Th, T helper; Breg, regulatory B.

## Discussion

Cellular immunity, humoral immunity, and complement are all involved in the CIDP pathogenesis. Although no specific antibodies have been found except for autoimmune nodopathies, antibody-mediated pathogenic mechanisms are widely recognized in CIDP. Strategies to reduce peripheral autoantibody levels or achieve B-cell depletion are considered promising (16, 22, 23). Efgartigimod, in comparison to IVIg and plasma exchange, has shown a more pronounced effect in decreasing serum IgG levels (8). The latest findings from the ADHERE study revealed that during the open-label phase, 66.5% of patients receiving efgartigimod treatment showed confirmed clinical improvement. In the randomized double-blind phase, the efgartigimod group demonstrated a significant 61% reduction in the risk of relapse compared to the placebo group, highlighting the substantial potential of efgartigimod in treating CIDP (24). In this case series, we investigated the efficacy and safety of efgartigimod in CIDP, addressing the real-world experience of the application of this therapy.

The study supports the efficacy of efgartigimod for treating CIDP, with a response rate of 100% and an effectiveness rate of 80% among the five patients. Notably, four of the five patients showed clinical score improvements within the first week of treatment, and clinical effectiveness was achieved by the fourth week in all four patients. These findings are consistent with those of the ADHERE study, in which participants demonstrated evidence of clinical improvement by week 4 and the time to first recorded improvement in the 25th percentile was 9 days. MG patients in the ADAPT study also exhibited significant score improvements in the first week, with the most pronounced improvements observed in the fourth and fifth weeks (9). Similarly, in an animal model of immune-mediated necrotizing myopathies (IMNMs), Julien et al. observed significant improvements in muscle strength on day 7 of efgartigimod treatment (25). These findings collectively suggest that efgartigimod can rapidly ameliorate the clinical symptoms of immune-mediated neuromuscular disorders. Given its rapid onset of action, efgartigimod holds promise as an effective rescue therapy for patients with immune-related neuromuscular diseases.

Patients 3, 4, and 5 were diagnosed with refractory CIDP and showed varying degrees of clinical improvement following efgartigimod treatment. Among them, patients 3 and 4 had previously received semi-annual low-dose rituximab treatments but still experienced disease progression. Patients 1 to 4 were undergoing treatment with other maintenance medications while receiving efgartigimod. However, following the addition of efgartigimod, their symptoms showed continued improvement. Accordingly, efgartigimod might be beneficial if added to maintenance treatment.

The present study employed a multidimensional set of assessment tools, including the INCAT and IRODS for evaluating disability; the MRC, grip strength, and the NIS for impairment assessment; and the TUG for gait disturbance evaluation (14). All five patients showed improvement on different scales. The patients with relatively milder symptoms and better baseline scores (Patients 1, 2, and 3) showed improvements in both impairment- and disability-related evaluation scales. In contrast, no significant changes in disability-related scores were observed in patients 4 and 5, who had more severe symptoms and worse baseline scores. Particularly in patient 4, who had the longest disease duration and the most severe axonal damage as illustrated by electrophysiological tests, clinical effectiveness criteria were not met. We hypothesize that the lack of clinical improvement is associated with severe secondary axonal damage due to demyelination. Therefore, for patients with more severe symptoms and worse scores, short-term evaluation of disability levels may be insufficient and long-term treatment and observation may be necessary.

The CIDP patients in this study demonstrated good tolerance of efgartigimod. Only Patient 1 reported diarrhea after the second dose, which was accompanied by a slight increase in white blood cell count. Apart from this, no patients reported any discomfort, including headache, infusion reaction, or infection. The overall adverse reaction rate was lower than that reported in previous studies (8–10). All five patients experienced varying degrees of IgG reduction during treatment, with the maximum reduction reaching 65% of the baseline, similar to previous reports in healthy individuals, the ADAPT study, and the ADHERE study (8, 9). The extent of IgG reduction did not show a clear correlation with the degree of clinical improvement. Previous reports suggested that a decrease in serum albumin might be an adverse reaction to FcRn inhibitors (26), but we did not observe such a phenomenon in the present study. On the contrary, we observed a slight short-term increase in serum albumin levels in the patients, although the reasons for this are unclear.

Flow cytometric analysis for three patients before and after efgartigimod treatment revealed a redistribution of T- and B-cell subpopulations in circulation. The treatment reduced the proportions of Th17 cells, memory B cells, and switched memory B cells, while increasing naive B cells and plasma cells. Previous study has also suggested an increase in Th17 cells in active CIDP (27). Similar trends have been observed with IVIg treatment, which also decreases memory B and switched memory B cells and increases naïve B cells (28). Apart from IgG trafficking and recycling, FcRn is also involved in antigen presentation, by dendritic cells and macrophages to CD4+ T cells, and by dendritic cells to CD8+ T cells. The combination of high levels of autoantibodies and subsequent Antibody-Dependent Cellular Cytotoxicity (ADCC), along with CD4+ T cell activity, can further augment humoral immunity by promoting the differentiation of B cells into autoantibody-secreting plasma cells and memory cells, thus leading to excessive immune responses (6, 29). Serum cytokines were measured in three patients as well, no notable changes were observed. These results indicate that, in addition to lowering IgG levels, efgartigimod may broadly regulate immune responses. Further studies with larger cohorts are needed to clarify the mechanisms involved.

This study has several limitations. The small size of the patient cohort leads to an inherent risk of selection bias. The majority of patients were typical CIDP, which may lead to a bias in the assessment of efficacy. Non-uniform treatment regimens make the observation of efficacy more challenging. The short follow-up duration impacts the observation of the long-term safety, efficacy and sustainability of therapeutic effects following the discontinuation of efgartigimod. Future controlled studies are needed that include a larger sample, a standardized protocol, and a longer follow-up. In addition, further research is needed to determine whether the intravenous formulation we used has similar efficacy to the subcutaneous formulation.

In conclusion, efgartigimod demonstrated promising effectiveness and safety in the short-term treatment of CIDP, offering a potential alternative therapy for patients who experience a limited response to standard of care. This study provides valuable insights into the real-world application of efgartigimod in CIDP and provides motivation for further studies of the effectiveness of efgartigimod for CIDP treatment.

## Data Availability

The original contributions presented in the study are included in the article/**Supplementary Material**. Further inquiries can be directed to the corresponding author.
