# Supplementary material for: Short-term treatment of CIDP with efgartigimod: a case series in China
Source: Front Immunol. 2025 May 1;16:1533167. doi: 10.3389/fimmu.2025.1533167 (PMC12078319; doi:10.3389/fimmu.2025.1533167)
Supplement: Supplementary file 1 [file Table1.docx]

Supplementary Table. Changes in clinical scores of CIDP patients

|  | Start（n=5） | Last（n=5） |
| --- | --- | --- |
| **INCAT** | 4.80±3.42(2-9) | 3.80±4.32(0-9) |
| **IRODS** | 23.00±21.44(0-43) | 28.00±25.13(0-48) |
| **NIS** | 92.70±70.25(20-180) | 76.80±76.48(15-175) |
| **MRC** | 38.20±5.06(0-60) | 43.40±21.14(7-60) |
| **Grip strength (rightt, kPa)** | 25.47±30.55(0.00-71.00) | 39.89±37.30(0.00-76.00) |
| **Grip strength (left, kPa)** | 26.74±30.94(0.00-73.30) | 36.46±35.64(0.00-72.00) |

Note: Data are mean±SD(range). “Start” indicates the timepoint of the initial administration of efgartigimod, and “Last” indicates the timepoint of last follow-up. INCAT, Inflammatory Neuropathy Cause and Treatment disability scale; IRODS, Inflammatory Rasch-built Overall Disability Scale; MRC, Medical Research Council sum score; NIS, Neuropathy Impairment Score.
